# Rainfall–Runoff Simulations to Assess the Potential of SuDS for Mitigating Flooding in Highly Urbanized Catchments

**DOI:** 10.3390/ijerph13010149

**Published:** 2016-01-21

**Authors:** Daniel Jato-Espino, Susanne M. Charlesworth, Joseba R. Bayon, Frank Warwick

**Affiliations:** 1 GITECO Research Group, Universidad de Cantabria, Av. de los Castros S/N, 39005 Santander, Spain; 2 Centre for Agroecology, Water and Resilience (CAWR), Coventry University, West Midlands CV1 5FB, UK; apx119@coventry.ac.uk; 3 Department of Project and Construction Management, Donostia City Council, Calle Larramendi 16, 20006 Donostia (Gipuzkoa), Spain; Joseba_Rodriguez@donostia.org; 4 Department of Geography, Environment and Disaster Management, Coventry University, West Midlands CV1 5LW, UK; aa4510@coventry.ac.uk

**Keywords:** geographic information system, rainfall–runoff simulations, stormwater modeling, sustainable urban drainage systems

## Abstract

Sustainable Urban Drainage Systems (SuDS) constitute an alternative to conventional drainage when managing stormwater in cities, reducing the impact of urbanization by decreasing the amount of runoff generated by a rainfall event. This paper shows the potential benefits of installing different types of SuDS in preventing flooding in comparison with the common urban drainage strategies consisting of sewer networks of manholes and pipes. The impact of these systems on urban water was studied using Geographic Information Systems (GIS), which are useful tools when both delineating catchments and parameterizing the elements that define a stormwater drainage system. Taking these GIS-based data as inputs, a series of rainfall–runoff simulations were run in a real catchment located in the city of Donostia (Northern Spain) using stormwater computer models, in order to compare the flow rates and depths produced by a design storm before and after installing SuDS. The proposed methodology overcomes the lack of precision found in former GIS-based stormwater approaches when dealing with the modeling of highly urbanized catchments, while the results demonstrated the usefulness of these systems in reducing the volume of water generated after a rainfall event and their ability to prevent localized flooding and surcharges along the sewer network.

## 1. Introduction

The global trends of urbanization in the 21st century are resulting in water being conveyed and discharged more rapidly than in its natural condition [1]. This entails an alteration of hydrological patterns and a growing flooding risk due to increased runoff volumes and decreased infiltration. The common practice to address this issue has habitually consisted of building conventional drainage infrastructures to minimize runoff accumulation. The water captured in this way is then transferred to an underground drainage network formed of a series of pipes and manholes connected to each other. However, depending on the amount of water they receive, these sewer systems often lack sufficient capacity to properly route their inflows, which might eventually result in localized floods and surcharges along the network.

The traditional approach to deal with this situation consists of oversizing and/or expanding the existing drainage infrastructures, which merely serves to delay the problem but does not provide a sustainable long-term solution. Alternatives to this course of action are Sustainable Urban Drainage Systems (SuDS), a series of techniques seeking to mimic the natural water cycle [2,3,4,5]. Their purpose is to mitigate runoff peak flow rates and reduce water pollution through infiltration, transport and retention mechanisms [6]. Sustainable drainage systems can also provide diverse benefits to public health. As pointed by Hellström *et al.* [7], these techniques can clean wastewater to avoid unhygienic conditions for users and mitigate stormwater to prevent damage derived from flooding. Green SuDS have been recommended because of the increase in evapotranspiration they involve, which has a positive impact on microclimates in urban areas, a factor of great importance to the health of urban dwellers [8,9]. Charlesworth [10] showed that SuDS also involve multiple benefits in the context of Climate Change for human and environmental health by carbon sequestration and storage, mitigation of the urban heat island effect and urban cooling. Green roofs and pervious pavements are two types of SuDS that are especially easy to integrate in cities, because they can be located in typical urban places such as buildings and parking areas.

Since drainage networks and urban catchments are space-dependent, the integration of Geographic Information Systems (GIS) with stormwater models provides an opportunity to simulate the impact of SuDS on urban drainage. Several authors have previously carried out different studies coupling both tools. Moore *et al.* [11] developed a GIS-based methodology to assist the selection procedure of stormwater disconnection opportunities from roofs, car parks and roads. Viavattene and Ellis [12] studied the effectiveness of a sustainable drainage system consisting of combining green roof, porous paving and infiltration trenches through an integration of 1D/2D modeling. Guan *et al.* [13] analyzed the influence of urbanization on drainage and then simulated the effect of different SuDS on the hydrological response of a catchment area. However, none of these studies succeeded in developing a comprehensive and accessible methodology to model the impact of SuDS at the spatial scale of a highly urbanized catchment. The first two did not discuss in detail how to introduce the sewer network in the subcatchment delineation process, while the latter took place in a mainly pervious catchment, where the infiltration mechanisms differ substantially from those that might exist in an almost completely impermeable area.

Under these premises, the aim of this research is to develop a thorough GIS-based stormwater simulation methodology to demonstrate the potential of SuDS for mitigating flooding in highly urbanized areas. The integration of two tools: ArcGIS [14] and the Storm Water Management Model (SWMM) [15], was proposed to achieve that purpose. The usefulness of the methodology was tested through a case study of a real catchment located in Donostia (Northern Spain), where the efficiency of two different green roofs and a pervious pavement structure for volume reduction was analyzed and compared with the performance of the existing drainage system. Although this particular area does not capture all the diversity that might exist in a whole city, it is a suitable location to prove the benefits that SuDS might involve when managing urban stormwater, because it is a highly urbanized catchment and contains feasible sites wherein to install these systems. In fact, the extension of this kind of analysis to larger areas would not provide an overall perspective of the impact of SuDS on urban drainage, but a collection of separate views corresponding to different catchments flowing to different places under different circumstances. The philosophy and methods proposed in this paper are independent of the specifics of the study site and can be applied to catchments with different sizes, degrees of imperviousness and feasible sites to implement SuDS.

## 2. Methodology

**Figure 1:**
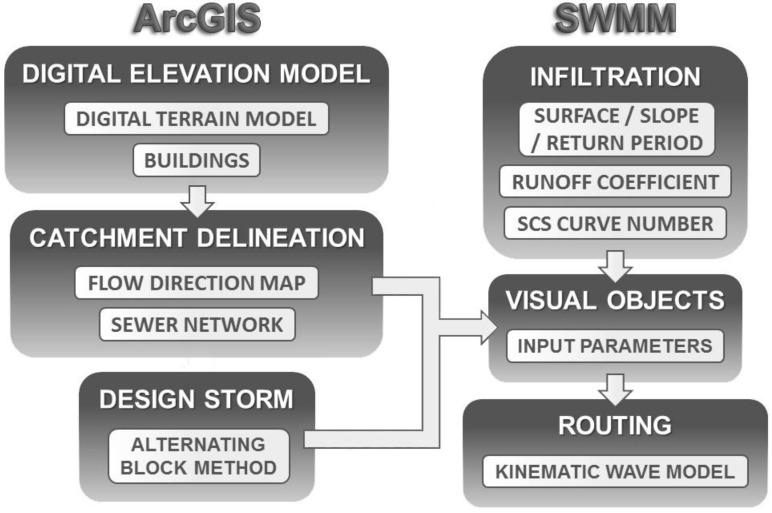
Graphical summary of the steps forming the proposed methodology.

The methodology on which this research was based consisted of two main modules, each of which was related to the use of the two tools proposed: GIS and stormwater models. Each module was based in turn on four main operations, as shown in Figure 1.

The first module was fully developed using ArcGIS 10.1, especially through its toolbar Arc Hydro [16]. GIS-based editing tools and zonal statistics were also applied to map infiltration areas according to their degree of imperviousness. The infiltration and routing processes were then simulated using SWMM 5.1 [17]. A synthetic storm was also designed using the Alternating Block Method [18] from the Annual Maximum Daily Precipitation (AMDP) data series corresponding to the study area.

The following subsections explain the theoretical foundations underlying each of the eight operations shown in Figure 1.

### 2.1. Digital Elevation Model (DEM)

The need for a Digital Elevation Model (DEM) arises from one question: how does urbanization change a catchment? On the one hand, it results in increased imperviousness of the ground surface (increased runoff) and decreased evapotranspiration (decreased vegetation). On the other hand, it implies the presence of barriers to natural water flow, such as buildings and sewerage.

In this context, a DEM is required to represent the landform of an urban area. In the absence of a reliable DEM, a methodology to produce such data from a Digital Terrain Model (DTM) and a shapefile containing the location of buildings was proposed. This process consisted of setting a positive elevation value in the polygonal vector layer of buildings to force surface water to flow around these obstacles. Then, the layer was converted to raster format and superimposed on a DTM, in order to include these barriers in the digital model. The DEM obtained after applying this procedure could be considered more rigorous than LiDAR-based DEMs in terms of horizontal accuracy, since the latter are very susceptible to registering noisy data. In contrast, vector layers are more reliable and easy to update.

### 2.2. Catchment Delineation

Catchment areas can be delineated from two inputs: the flow direction map and a stream network defined according to the cells in which flow accumulates. Flow direction was determined from the DEM using the eight direction (D8) flow model proposed by Jenson and Domingue [19]. Since this research focuses on urban areas, such a stream network was represented by the existing sewer network. The size of the catchment area to be studied was thus determined by the drainage network itself, which was therefore the limiting factor in its geometrical arrangement. The catchment so identified was independent from neighboring ones, which flow to different places, leading to each of them having to be studied independently. This layer was revised before use to identify and clean up possible loops, dummy nodes and intersections throughout its geometry. After that, the sewer network became a polyline segmented according to the location of the manholes. The polyline was rasterized and then combined with the flow direction map; catchment areas were delineated such that water was forced to be captured and conveyed along the sewer network.

### 2.3. Design Storm

When applied to urban areas, storm duration is often assumed as the time of concentration of the whole catchment [20], a value equal to 60% of its lag time, according to the Soil Conservation Service (SCS) [21]. The lag time ($T_{L}$) is a function of the slope (Y), length (L) and retention capacity of the catchment (S) (see Equation (1)) which are easily calculable parameters using GIS tools.

(1) $$T_{L}=L^{0.8}\;\times \;\frac{{\left(S+1\right)}^{0.7}}{1900\;\times \;\sqrt{Y}}$$

A rainfall event can be simulated by designing a synthetic storm using the Alternating Block Method from daily rainfall data recorded over a long time period. Some authors state that this period must be at least 20 or 30 years [22,23]. The first step to design a synthetic storm is to obtain the maximum daily rainfall for a certain return period using the probability distributions that best fit the patterns of such precipitation measurements. Furthermore, the IDF (Intensity–Duration–Frequency) curves associated with this daily precipitation can be calculated to obtain the rainfall intensity of the design storm. In line with the recommendations found in the literature regarding the modeling of urban catchments [24,25,26,27], three different return periods (2, 5 and 10 years) were proposed for these calculations.

From there, the Alternating Block Method defines the synthetic design storm from pairs of Intensity–Duration values. It calculates values of precipitation corresponding to a series of n intervals of intensity $I_{\Delta t}$ and duration Δt, such that D=n × Δt. These values are arranged in blocks alternately around the interval with the highest precipitation to create a hyetograph representing the storm event.

### 2.4. Infiltration

Land use maps, which are often employed to model infiltration [28,29], are another type of data that can lack sufficient resolution and update. For example, the standard CORINE Land Cover (CLC) maps, widely used across Europe, are developed at a scale of 1:100,000, while the later version (2012) is only available for some countries. Given the common magnitude of urban catchments, a more accurate representation of the infiltration parameters of the study area was required.

Chow *et al.* [18] provided a series of values to determine the runoff coefficient of urban areas from three inputs: return period, slope and surface type. Slope can be easily computed using GIS resources from a digital model representing the elevation of the study area. Ortophotos, which are normally available at high resolution (below 50 cm), can be used as the basis for delineation of different surface types and therefore to determine what the composition of the catchment areas is in this sense. From these two operations and a given return period, obtaining the runoff coefficient of a catchment is automatic.

Included in SWMM, the Soil Conservation Service (SCS) Curve Number Method [30] was the most suitable model to characterize infiltration. Unlike Horton and Green–Ampt models, which are based on difficult parameters to define in urbanized areas such as infiltration and moisture conditions of the ground, the SCS Curve Number Method is directly related to the runoff coefficient (*C*) through the curve number (*CN*) (see Equations (2) and (3)), an empirical parameter used to predict infiltration from rainfall excess.

(2) $$C=\frac{\left[\left(P_{d}/P_{o}\right)-1\right]\;\times \;\left[\left(P_{d}/P_{o}\right)+23\right]}{{\left[\left(P_{d}/P_{o}\right)+11\right]}^{2}}$$

(3) $$CN=\frac{25400}{254+\frac{P_{0}}{0.2}}$$

where *P_d_* is the average precipitation of the study area and *P_o_* is the runoff threshold, a coefficient indicating the spatial variation in soil moisture at the beginning of the rainfall event. For a given subcatchment, the calculation of this coefficient can be determined by solving Equation (3) from its runoff coefficient and precipitation. From there, the curve number, which is the parameter required by SWMM to model infiltration, is determined through Equation (2). 

### 2.5. Visual Objects

Visual objects are those required by SWMM to represent a stormwater drainage system. Four different types of objects were necessary for modeling the conditions to be simulated in this study: subcatchments, LID Controls (SuDS), conduits and nodes.

As for the subcatchments, up to 9 different parameters were defined to properly characterize them. Some, such as Area, Slope and % of Imperviousness, can be determined using GIS-based editing and zonal statistics tools. Slope in each subcatchment can be obtained from averaging the slope in each of its cells, previously obtained from the DEM. Similarly, the delineation of both impervious and pervious areas from the ortophoto and the subsequent calculation of their proportion in relation to the subcatchment area enables the degree of imperviousness to be determined. Width was another geometric parameter that was estimated as the subcatchment area divided by the average length of the flow paths from the furthest drainage points of the subcatchment [17]. The remaining parameters were related to the Manning’s roughness coefficient and depth of depression storage of the impervious and pervious areas forming the subcatchment. Typical values for these parameters can be found in McCuen *et al.* [31] and ASCE [32], respectively.

SWMM’s LID Control Editor allows eight different types of SuDS to be modeled by characterizing the layers and materials forming them. Reference values for these parameters can be found in SuDS-related specialized literature [33,34] and previous studies on the use of SWMM for modeling the impact of these systems on urban drainage [35,36,37,38].

With respect to the sewer network, manholes were introduced in the model through their elevation and depth, while the inputs required to define the pipes were their diameter, length and roughness [39]. SWMM uses Manning’s equation (see Equation (4)) to set the relationship between flow rate (*Q*), cross-sectional area (*A*), hydraulic radius (*R_h_*) and slope (*Y*) throughout the pipelines. For I.S. units:

(4) Q=A × Rh23 × Yn

where n is the Manning’s roughness coefficient. Although some of the parameters of the sewer network can be determined using a GIS, they were all specified in the original data layer.

### 2.6. Routing

Flow routing throughout a pipeline is governed by the conservation of mass and momentum equations for both gradually varied flow and unsteady flow (*i.e.*, the Saint Venant equations) [17]. Three different models are available in SWMM to solve these equations, from less to more complex: steady flow, kinematic wave and dynamic wave routing.

The choice of routing model depends on the purpose of the simulation. Since the focus of this study was the potential of SuDS for attenuating flooding risk, the model must show a balance between being conservative but not too simplistic. For this reason, the kinematic wave model was proposed to solve these equations.

This model solved the continuity equation and a simplified form of the momentum equation in each pipeline forming the sewer network [17]. Such simplification consisted of considering that the slope of the water surface and the conduit were equal. Thus, each conduit conveyed flow up to its maximum according to Manning’s equation (see Equation (4)). Excess flows were either lost from the model or ponded on top of the inlet node and then routed again when capacity was available.

## 3. Results and Discussion: A Case Study in Donostia, Spain

In order to evaluate the impact of SuDS on urban drainage, the methodology described above was applied to a highly urbanized catchment located in the southeastern part of Donostia (Northern Spain). This is one of the rainiest cities in Spain, with an average annual precipitation around 1500 mm/year [40], which makes it suitable to assess the potential of SuDS for attenuating flooding.

The first two datasets required to delineate the subcatchments forming the catchment area were collected from GeoEuskadi, the Spatial Data Infrastructure (SDI) of the Basque Country [41]. They consisted of a DTM in raster format with a cell size of 1 m and a polygonal shapefile of buildings at a scale of 1:5000. The DTM was built from data retrieved from a LiDAR survey with a resolution of 0.5 points/m^2^. It describes the bare soil surface on the Geodetic Reference System ETRS89 through its orthometric heights, which are determined using the EGM08-REDNAP geoid model with an absolute accuracy of 3.8 cm [42]. The DTM is provided by sheets through a 5000 m mesh size in .asc format according to the Aerial Ortophotography Spanish Plan. A CAD file containing the features of the sewer network across the whole city was supplied by Donostia City Council [43] at a scale of 1:5000, including the diameters, lengths, depths and materials, characterizing both the pipes and the manholes forming it.

**Figure 2:**
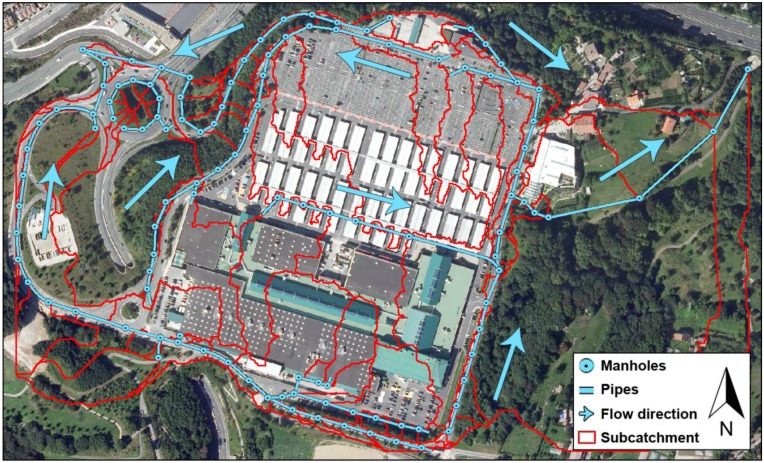
Catchment area under study and geometric arrangement of its portion of sewer network.

Following the steps explained in Subsections 0 to 0, this information was used to delineate the catchment, which covered 31.40 hectares. As a result of this process, up to 130 different subcatchments were identified as shown in Figure 2. The parameters required by SWMM to characterize each of these subcatchments were computed using ArcGIS statistical tools, while the features of the sewer network were available using the original CAD layer provided by the City Council. The portion of sewer network corresponding to the catchment under study consisted of 115 manholes and 113 pipes with diameters from 200 mm to 1000 mm.

According to the lag time of the catchment area (see Equation (1)), storm duration was set at 150 min. Both storm duration and central coordinates of the catchment area were used as inputs in the software MAXPLU (Maximum Daily Rainfall in Peninsular Spain) [44] to obtain the maximum daily rainfall corresponding to a 10-year return period. This software is based on a monograph prepared by the Spanish Ministry of Public Works in collaboration with the Spanish Meteorological Agency (AEMET) [45] and provides a direct alternative to the IDF curves. This consisted of the generation of several precipitation maps for different return periods from spatial interpolation of the data recorded by 1545 Spanish meteorological stations covering more than 30 years. The storms determined using the Alternating Block Method for the return periods of two and five years were insufficient to produce relevant drainage problems in the catchment. Consequently, the synthetic storm corresponding to the 10-year precipitation value and the rainfall intensity associated with it was used for calculations hereinafter.

**Figure 3:**
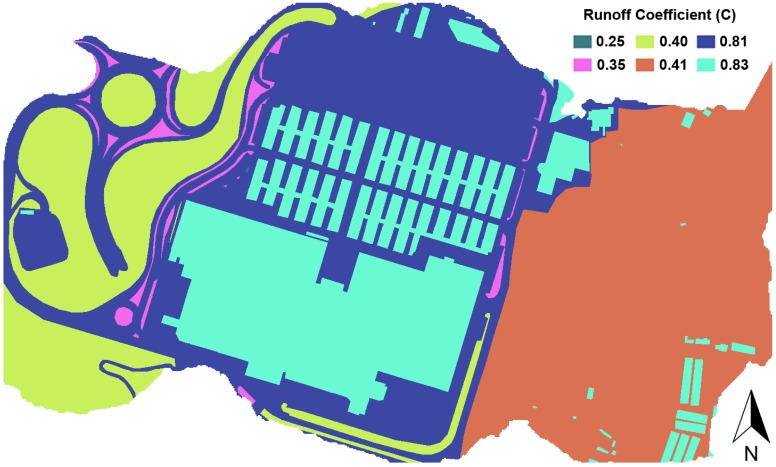
Runoff coefficients corresponding to the different surface types present in the workspace.

The ortophoto shown as a base layer in Figure 2, which was also acquired from GeoEuskadi with a cell size of 25 cm, was used to delineate the different surface types present in the study area in terms of infiltration. Figure 3 represents the set of runoff coefficients obtained according to such a return period and the surface types and slope values corresponding to the workspace [18]. Curve numbers were calculated using Equations (2) and (3) according to these runoff coefficients and the 10-year precipitation value for the catchment.

**Figure 4:**
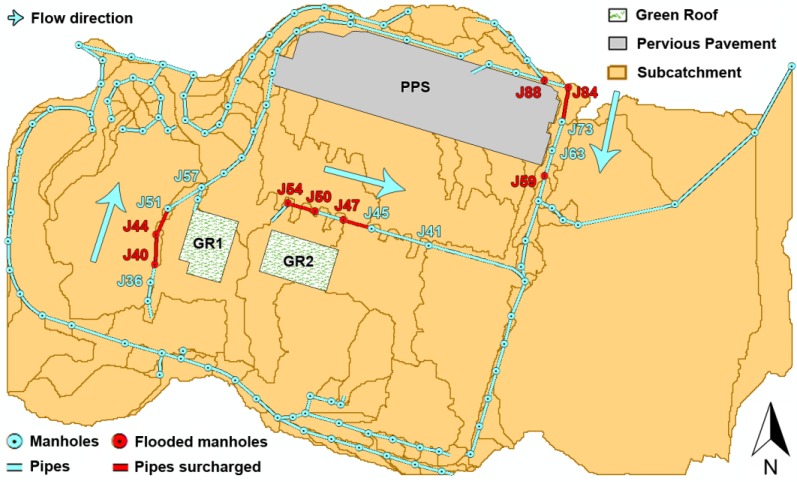
**Figure 4**. Layout of subcatchment areas, flooded nodes, pipes surcharged and Sustainable Urban Drainage Systems (SuDS).

The simulation duration was set at 4 h after checking that this value was time enough for runoff to completely disappear. Furthermore, the time steps for both reporting and routing were set at 3 s, because the continuity errors were reduced to almost negligible (less than 0.1% for both surface runoff and flow routing). Thus, the simulation of the designed storm event under existing drainage conditions was run, resulting in several flooded nodes and surcharged pipes (those highlighted in Figure 4). Each of these sewer network sections was connected to a series of almost completely impervious subcatchments where there is very little infiltration (see Figure 3), which explains their inability to manage their respective inflows.

Two different SuDS, two extensive green roofs (GR1 and GR2) and a porous asphalt pervious pavement structure (PPS), were included in the model to assess their storm attenuation capability. Each of them was located in a subcatchment connected to the flooded nodes (or junctions) and surcharged pipes and/or in any of the previous subcatchments to these conflictive elements.

The places selected for installing these SuDS are two roofs located in the west-central part of the workspace and a parking area in the north of the catchment area (see Figure 4). A summary of the parameters introduced in SWMM’s LID Editor to define both types of system is provided in Table 1. These values were derived from those suggested in a SuDS-related bibliography [33,34,35,36,37,38], in order to replicate the typical drainage systems that could be implemented in Northern Spain, according to raw materials availability and climatic conditions.

**Table 1:** Parameters introduced in the Stormwater Management Model Low Impact Development (SWMM’s LID) Editor to characterize both SuDS techniques.

SuDS	Layer	Parameter	Value
Green Roofs	Surface	Vegetation Volume Fraction	0.5
		Roughness (Manning’s n)	0.15
	Soil	Thickness (mm)	100
		Porosity (volume fraction)	0.5
		Field Capacity (volume fraction)	0.2
		Wilting Point (volume fraction)	0.1
		Conductivity (mm/h)	12.7
		Conductivity Slope	10
		Suction Head (mm)	88.9
	Drainage Mat	Thickness (mm)	10
		Void Fraction	0.75
		Roughness (Manning’s n)	0.03
Pervious Pavement Structure	Surface	Roughness (Manning’s n)	0.02
	Pavement	Thickness (mm)	100
		Void Ratio (Voids/Solids)	0.2
		Permeability (mm/h)	254
	Storage	Thickness (mm)	300
		Void Ratio (Voids/Solids)	0.6
		Seepage Rate (mm/h)	3.3

To test the influence of these systems to prevent flooding, three simulations were run, including each of the SuDS proposed. The aim at this point was to compare the behavior of the nodes and pipes highlighted in Figure 4 with and without SuDS installed.

**Figure 5:**
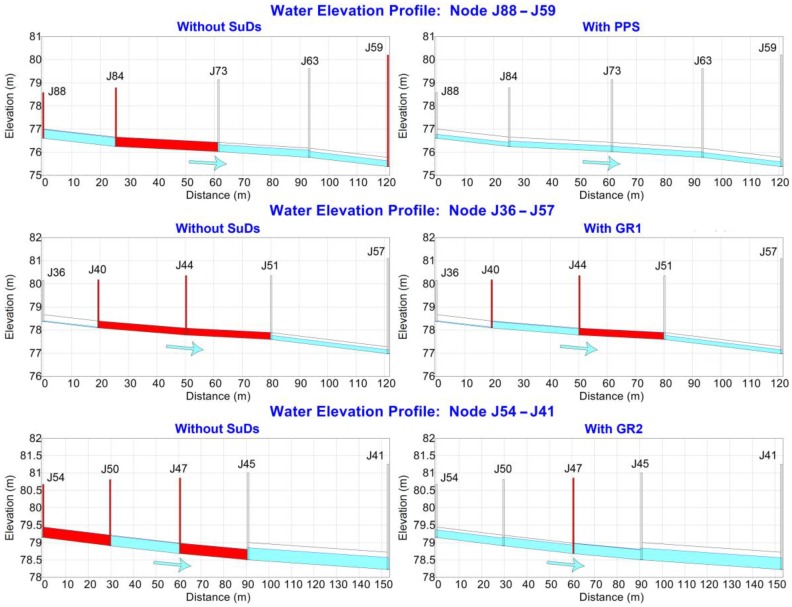
Water elevation profiles of the sewer network sections under analysis, before and after installing SuDS.

Figure 5 shows the profile plots of the sewer network sections containing the problematic nodes and pipes at their most critical time in terms of drainage. To a greater or lesser extent, the inclusion of SuDS proved to be positive when avoiding localized floods and surcharges. In fact, the results were gradual, since each of the systems reached different degrees of success, from GR1, which only was capable of preventing one of the pipes from surcharging, to PPS, whose presence resulted in the complete removal of both flooding and surcharges. The reason for these differences can be explained with the following variables: the location and area occupied by SuDS with respect to the catchment area corresponding to these sewer network sections and the geometrical arrangement of the sewer network sections themselves. Figure 4 and Figure 5 show that the effect of PPS began from node J88 and covered a large proportion of the area flowing to it. This section also showed a progressive increase in manhole depth and a slope steep enough to circulate water smoothly. In contrast, the central part of Section J36–J57 was rather flat and the diameter of the pipes too small to deal with the inflow, despite the presence of GR1. These issues were solved almost completely in the case of GR2, whose impact resulted in only one node remaining flooded.

**Figure 6:**
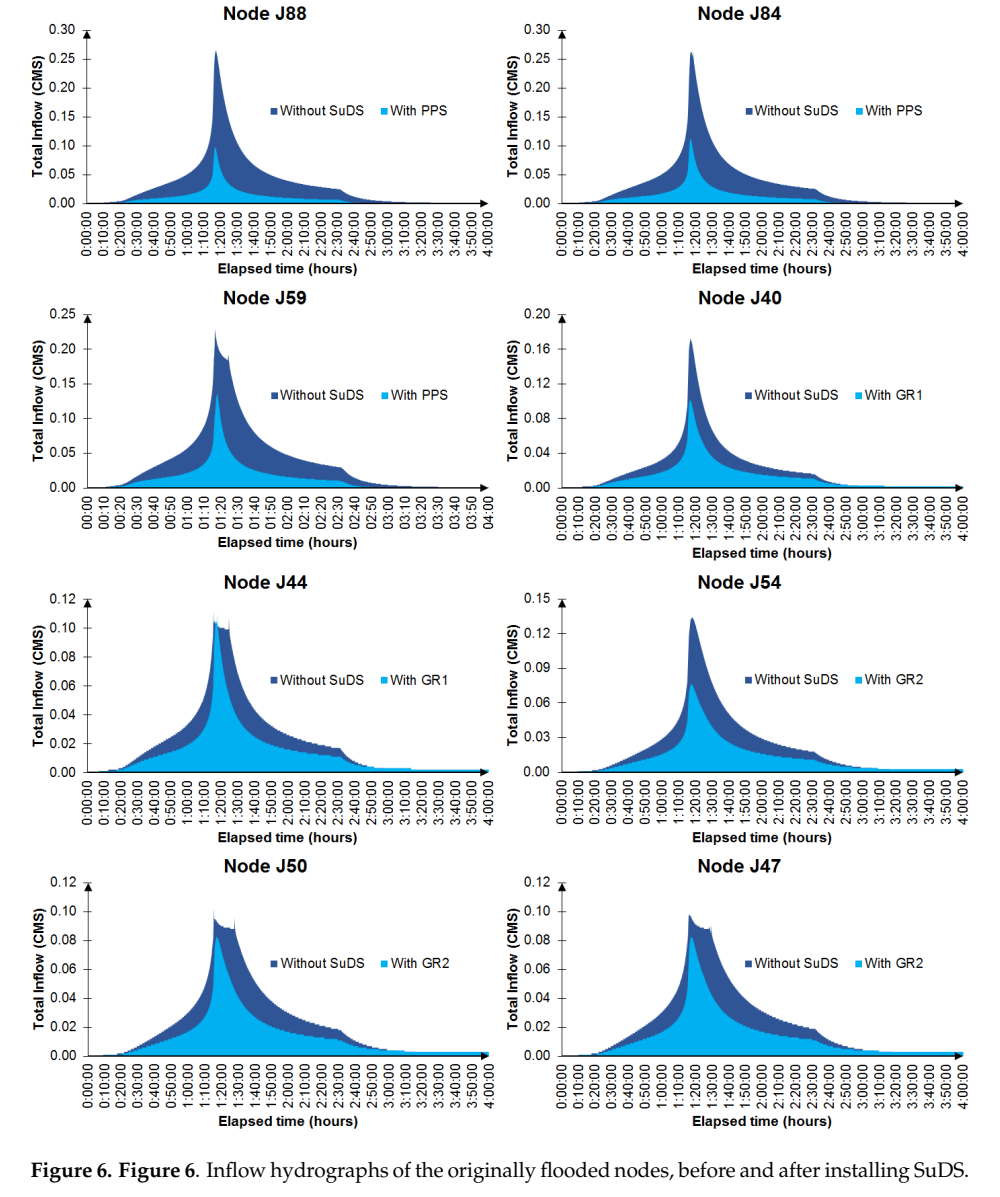
**Figure 6**. Inflow hydrographs of the originally flooded nodes, before and after installing SuDS.

The impact of SuDS on urban drainage was also analyzed according to the inflow received by the originally flooded nodes. Figure 6 shows the inflow hydrographs corresponding to the flooded nodes shown in Figure 4 and Figure 5 with and without SuDS. The inclusion of these systems resulted in an important reduction in total inflow generated in their respective nodes, even in the case of GR1, which showed the worst performance in terms of avoiding floods and surcharges. The double peak appearing in some of the hydrographs (J59, J44, J50 and J47) was due to the surcharge of one of the pipes before these nodes. In other words, the maximum velocity and discharge of a circular pipe is reached before it is totally full. The first peak was higher because it included the lateral inflow of the subcatchment associated with the node in question.

Table 2 summarizes the performance of the three SuDS in terms of their impact on the volume generated as a result of the storm event in the originally flooded nodes, before (*Vo*) and after (*V_SuDS_*) inclusion of these systems. Again, the volume reduction (*V_R_*) due to the PPS was notably higher than that of the green roofs, mainly due to its greater spatial extent in relation to the size of the subcatchment in which it is placed. In addition, the combined action of the pervious pavement layers has been proven to be more capable of reducing runoff volumes than those of a green roof [46]. Moreover, although the differences decreased, the PPS was also the most efficient for those simulated, with the best ratio achieved between volume reduction and area of SuDS required (*Eff.*). These values are consistent with those provided in previous studies of the performance of these types of SuDS in terms of volume reduction [33,47,48,49,50].

**Table 2:** Effectiveness of the three SuDS simulated for volume reduction.

SuDS	*Vo* (m^3^)	*V_SuDS_* (m^3^)	*V_R_* (%)	*Eff.* (m^3^/m^2^)
PPS	1519.0	492.8	67.6	0.138
GR1	610.4	382.9	37.3	0.094
GR2	936.6	567.8	39.4	0.116

***Vo*** = Volume before including SuDS; ***V_SuDS_*** = Volume after including SuDs; ***V_R_*** = Volume reduction; ***Eff.*** = Efficiency.

## 4. Conclusions

This paper presents a GIS-based stormwater simulation model to analyze the impact of SuDS on urban drainage after a rainfall event. For this purpose, an *ad hoc* methodology based on the combination of ArcGIS and SWMM was developed. The first was used to generate the information required to parameterize the subcatchments according to topography and sewer network in the workspace, while with the latter, a series of rainfall–runoff simulations were run to compare the current drainage system with other scenarios including SuDS.

The case study results proved the usefulness of these SuDS to mitigate stormwater impacts and help to avoid localized floods and surcharges along the sewer network. The correct design and placement of these systems for maximum efficiency in providing flood resilience is vital in order to address the adverse impacts of flooding and sewer surcharge on human health. Apart from their intrinsic infiltration capacity, the degree of effectiveness of these systems depended on their geometric relationship with the existing drainage network. Of the three cases tested in this study, that corresponding to the PPS covered a larger part of the inflowing catchment area and was better placed in relation to the origin of runoff. In addition, the water elevation profiles also showed that a poorly designed drainage network can significantly hinder the work of SuDS.

The process followed to reach these results demonstrated that the combination of a DEM, created manually from a DTM and a building vector layer, and a sewer network segmented according to the location of manholes, is a suitable and accessible method to delineate the subcatchments forming an urban catchment. Furthermore, the subcatchment characteristics required to run a rainfall–runoff simulation can also be easily computed using GIS-based editing tools and zonal statistics from a DEM and a layer containing the surface properties of the study area.

In summary, the integration of GIS tools and stormwater models highlights the opportunity to improve urban water resources management. Moreover, the LID editor of SWMM allows the definition of different SuDS techniques and the simulation of their impact on urban drainage, in order to compare the effectiveness of various stormwater strategies. In other words, the findings of this research can help to better design drainage plans which can restore the natural water cycle in urban areas as far as possible.
